# Mitochondria and Female Germline Stem Cells—A Mitochondrial DNA Perspective

**DOI:** 10.3390/cells8080852

**Published:** 2019-08-08

**Authors:** Justin C. St. John

**Affiliations:** The Mitochondrial Genetics Group, Robinson Research Institute, School of Medicine, Adelaide Health and Medical Sciences Building, The University of Adelaide, Adelaide, SA 5005, Australia; jus.stjohn@adelaide.edu.au

**Keywords:** mitochondria, mitochondrial DNA, mitochondrial DNA copy number, female germ cells, egg precursor cells, embryonic stem cells, DNA methylation, mtDNA set point

## Abstract

Mitochondria and mitochondrial DNA have important roles to play in development. In primordial germ cells, they progress from small numbers to populate the maturing oocyte with high numbers to support post-fertilization events. These processes take place under the control of significant changes in DNA methylation and other epigenetic modifiers, as well as changes to the DNA methylation status of the nuclear-encoded mitochondrial DNA replication factors. Consequently, the differentiating germ cell requires significant synchrony between the two genomes in order to ensure that they are fit for purpose. In this review, I examine these processes in the context of female germline stem cells that are isolated from the ovary and those derived from embryonic stem cells and reprogrammed somatic cells. Although our knowledge is limited in this respect, I provide predictions based on other cellular systems of what is expected and provide insight into how these cells could be used in clinical medicine.

## 1. Introduction

There is an increasing interest in the generation and use of female germline stem cells to study their properties and propensity to produce mature oocytes [1]; and to use them to treat infertile couples [2,3,4]. Consequently, there is a need to understand the roles that mitochondria and the mitochondrial genome play in development; and to further understand how the failure to establish synchrony between the nuclear and mitochondrial genomes could render these cells dysfunctional. In this review, I describe the processes that would affect female germline stem cells, whether isolated from the ovary, or derived from embryonic stem cells or induced pluripotent stem cells, and how their failure to act affects gamete quality and fertilization outcomes. Importantly, I demonstrate that the interactions of the nuclear and mitochondrial genomes are complex and appear to require their establishment from the very early stages of development to ensure that there would be no inherent complications passed onto the resultant offspring.

## 2. The Role of Mitochondria

Mitochondria play critical roles in cellular function and are found in nearly all mammalian cells. In adult cells, mitochondria tend to be mature, as they form highly structured networks that influence cellular function. They have the capability to store Ca^2+^ [5], as well as initiate other key biological processes such as steroidogenesis through the reduction of cholesterol [6]. They further act by balancing free radical activity to reduce excess free radicals that are not required for intracellular activities, and would, otherwise, affect cellular function [7]. They are regulators of apoptosis and necrosis [8], as well as innate immune responses to pathogens and cell stress [9,10,11]. Of increasing importance is their role as activators and regulators of the epigenome. In this instance, the by-products of the citric acid cycle, for example, α-Ketoglutarate, act as co-factors with the TET family of proteins to mediate the transition of 5-methylcytosine to 5-hydroxymethylcytosine, i.e., to convert methylated DNA to demethylated DNA [12]. Likewise, they can modulate the activity of histone modifiers [13]. Finally, they act as the vehicles for the propagation and transmission of the mitochondrial genome through the developing female organism and into subsequent generations [14] to maintain and ensure the maternal-only transmission of mtDNA.

In embryonic stem cells, adult stem cells, primordial germ cells, egg precursor cells, and maturing oocytes and embryos, mitochondria are naïve, transparent, roundish-oval-like structures, which have limited capacity to produce energy [15,16]. This is unlike their mature counterparts that are elongated, dense structures, which form complex networks to promote interactions between mitochondria [17]. Early, naïve cells also have few mitochondria and they achieve these complex networks by multiplying and, simultaneously, migrating from one end of the cell to surround the nucleus and populate the other end of the cell as they undergo differentiation into mature cell types [18]. They also initiate mitochondrial fusion through the expression of the Mitofusins (Mfn1 and 2) that promote the state of a mature mitochondrial continuum within a cell [19].

## 3. Energy Production

**Figure 1:**
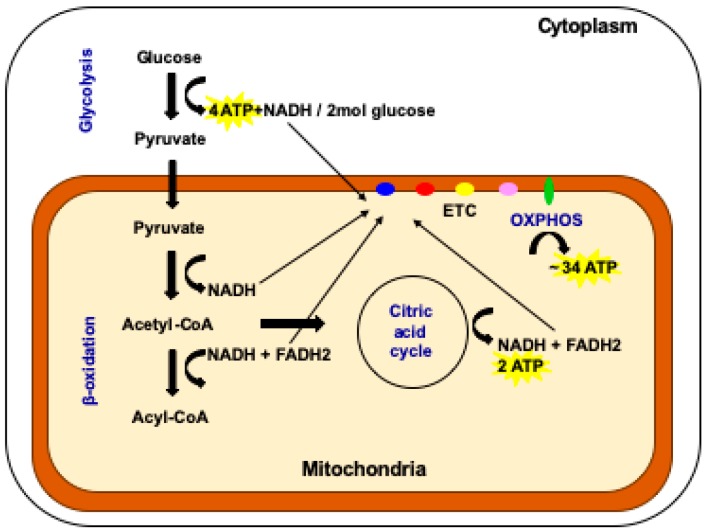
The multiple pathways available for the production of cellular ATP. In the cytoplasm of the cell, glycolysis generates four molecules of ATP for every two molecules of glucose invested. This is the pathway favored by fast replicating cells. In the mitochondrion, the processes of β-oxidation, the citric acid cycle and OXPHOS take place. OXPHOS is favored by cells with complex functions. Two molecules of ATP are generated by the citric acid cycle and 34 molecules are generated by OXPHOS (adapted from reference [20]).

There are several energy generating pathways in the cell (see Figure 1). One key pathway is glycolysis, which takes place in the cell’s cytoplasm [20]. It produces relatively low levels of ATP (4 mol for every 2 mol of glucose invested in the process) but is a highly efficient and rapid process and is favored by fast replicating cells that require energy quickly [20]. In mammalian systems, they would include cells that do not perform complex functions such as embryonic cells, embryonic stem cells, adult stem cells, tumor-initiating cells, and female germ cells from various stages of development up to and including mature oocytes. Blood cells also rely on glycolysis as they are recruited in a rapid fashion to react to an insult [21].

Cells that have more complex functions, for example heart, muscle, and nerve cells, use the by-products of glycolysis that pass into the mitochondria [22,23] (Figure 1). Pathways that include β-oxidation and the citric acid cycle provide electrons that enter the electron transfer chain to produce the vast majority of cellular ATP, approximately 36 mol from 2 mol of glucose invested, through the biochemical process of oxidative phosphorylation (OXPHOS) (Figure 1) [20]. The electron transfer chain is located in the inner mitochondrial membrane and also establishes the mitochondrial membrane potential that provides the mitochondrion with a barrier to regulate the import and export of factors and protect its integrity and the viability of the cell [5,15]. In cells of a more complex nature, the folding of the inner membrane results in cristae that provide a denser, more opaque nature and is indicative of their higher capacity for OXPHOS [24]. Unlike any other cellular apparatus in the cell, the electron transfer chain is encoded by two separate genomes: the nuclear and the mitochondrial genomes.

## 4. The Mitochondrial Genome

The mammalian mitochondrial genome ranges from 16.2kb (mouse) [25] to 16.7kb (pig) [26] in size. It encodes 13 of the approximate 100 subunits of the electron transfer chain, 22 tRNAs and 2 rRNAs. It has one major non-coding region, the Displacement or D-loop, which comprises two hypervariable regions indicative of an individual’s maternal ancestry; and the control region, which contains regulatory sites for the initiation of transcription and replication. The control region is also the site of interaction for the nuclear-encoded transcription and replication factors that translocate to the mitochondrion to drive these events. mtDNA copy number is cell type specific and relates to the ATP requirements of that particular cell for OXPHOS-derived ATP [23]. 

## 5. The Regulation of mtDNA Copy Number during Development

**Figure 2:**
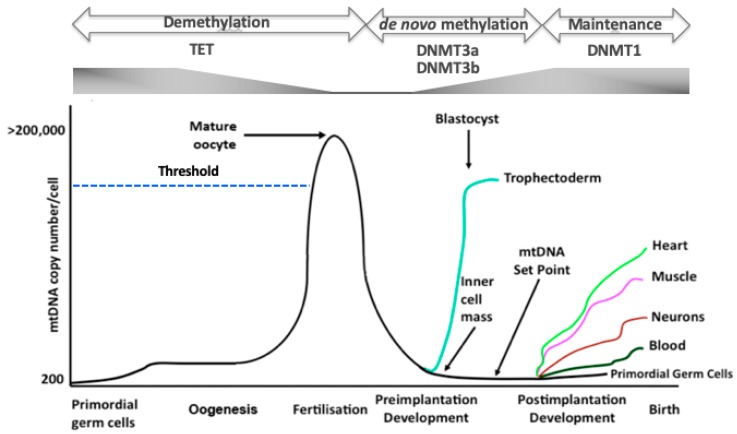
The regulation of mtDNA copy number during development. In primordial germ cells, mtDNA is maintained at low levels. As oogenesis progresses, mtDNA copy number increases significantly and is then arrested at the metaphase II stage. A threshold (broken blue line) needs to be reached in order that oocytes mature and fertilize. Following fertilization, mtDNA copy number decreases through to the blastocyst stage. mtDNA replication is initiated in the trophectoderm, whilst the ICM continues to reduce mtDNA copy number. This enables the developing embryo to establish the mtDNA set point prior to differentiation. Following commitment to a specific lineage, cells then replicate their mtDNA in a cell-specific manner to enable them to perform their specialized functions through OXPHOS, as required. Furthermore, there are synchronous changes to DNA methylation and gene expression profiles throughout these processes. TET enzymes reduce parental DNA methylation through to the blastocyst stage whilst de novo DNA methylation, mediated by DNMT3a and DNMT3b, is initiated in the blastocyst. DNMT1 then maintains the newly established cell-specific DNA methylation profiles.

mtDNA copy number is strictly regulated during development. The primordial germ cells, the very first germ cells to be laid down following migration into the ovary [27,28], have very low levels of mtDNA, approximately 200 copies per cell [29,30,31]. These copies then form the template for all mtDNA that is transmitted through the germline; and, during oogenesis, are exponentially replicated (Figure 2) [29,30,32]. As mitochondria maintain their immature status during oogenesis, they do not contribute extensively to the generation of ATP. Consequently, the naivety of these mitochondria and the high mtDNA copy number present in the mature, metaphase II oocyte, is regarded as an investment in subsequent developmental events as, just prior to this stage, the last mtDNA replication event takes place until post-gastrulation in the embryo proper [33]. This counters the argument that mitochondria have a key role to play in ‘fueling oocyte activity’ [34] and supports the view that mitochondria in very early development maintain cell viability by having efficient membrane potentials generated by protons passing from the electron transfer chain into the mitochondrial inner membrane space [35,36]; and that loss of OXPHOS does not prevent germ cell differentiation [37]. Consequently, oocyte mitochondria act as vehicles for the transmission of the mitochondrial genome. Between the germinal vesicle and the mature metaphase II stages, there are a number of refining mtDNA replication events that likely act as purifying processes prior to the metaphase II stage, which contains the mtDNA available for transmission through to the offspring [38].

During preimplantation development, as there is no replication of mtDNA, when each cell divides the mtDNA content of each newly formed embryonic cell is considerably reduced [38] (Figure 2). Indeed, there appears to be an active process to extrude mtDNA from the embryo during preimplantation development, as mtDNA from the embryo is found in its neighbouring environment [39]. The blastocyst, which represents the first differentiation and compartmentalisation events in the embryo, mediated by lineage specific gene expression [40] and metabolic changes [41], initiates mtDNA replication in the trophectodermal cells [38]. On the other hand, the cells of the inner cell mass continue to dilute out their mtDNA content as they divide [38] and, just prior to gastrulation, establish the ‘mtDNA set point’ [42,43,44]. 

The mtDNA set point contains the founder population of mtDNA that contributes to the primordial germ cells of the next generation and to the somatic tissues of the offspring (Figure 2). The mtDNA set point contains very few copies of mtDNA per cell (< 100 copies). This template not only contributes to the primordial germ cells of the next generation but also restricts the units of mtDNA inheritance to approximately 200 copies through a recycling process that exists to maintain the strict maternal inheritance of mtDNA and defines the molecules of mtDNA contributing to an individual’s maternal ancestral lineage. Indeed, this might be a defining stage in development that could account for the inclusion or rejection of sperm mtDNA that has bypassed the tight elimination process [45,46,47], which takes places prior to embryonic genome activation [48], although this form of biparental transmission tends to be a very rare event [45]. Furthermore, this template is used by the somatic tissues and is replicated in a cell specific manner so that cells giving rise to complex functions such as heart, muscle, and brain cells acquire the high numbers of mtDNA copy they require to perform their complex functions and those that primarily rely on glycolysis acquire considerably fewer copies (Figure 2).

## 6. mtDNA Replication Events Are under the Control of the Nuclear Genome

One of the key attributes to mtDNA replication is that the process is under the control of a number of genes encoded by the nuclear genome, which combine with the mitochondrial genome to form nucleoids [49,50]. Indeed, this complex process is tightly coupled to mitochondrial biogenesis in that a number of these factors appear to be precursors or upstream regulators of mtDNA replication [51]. These include STAT3, HIF1α, SIRT1, MYC, PGC1α/β, NRF1/2, ERRα/β/γ, SIRT3, and PPARα/β. However, the key interactors with the mitochondrial genome are the mitochondrial single-stranded-binding protein (mtSSB); the mitochondrial-specific helicase Twinkle, the catalytic subunit of the mitochondrial-specific polymerase (POLGA) and its accessory subunit (POLGB); and mitochondrial-specific DNA topoisomerase I (TOP1MT) [49,51]. Not only does there need to be a high degree of compatibility between the two genomes to ensure that the subunits of the electron transport chain function effectively together, but also the presence and persistence of mtDNA in the germline is reliant on the nuclear genome being able to transcribe and replicate the mitochondrial genome, especially during germ cell differentiation as mtDNA copy number increases considerably. Both facets are important to cellular function. 

## 7. The Relationship between DNA Methylation, mtDNA Replication and Oogenesis

Oogenesis is under the control of DNA methylation [52,53]. The later stages of oogenesis are associated with the reduction in DNA methylation [52,53] and consistent with increases in mtDNA copy number [29,30] (Figure 2). Consequently, it appears that the early female germ cells are initially extensively DNA methylated and then undergo epigenetic reprogramming with demethylation-resistant regions being enriched for repressive chromatin marks, for example H3K9me2/3, and regions that demethylate being enriched for active chromatin marks, namely H3K4me3 or H3K27ac [54]. They also exhibit low mtDNA copy numbers [29,30,31]. Indeed, this is not dissimilar to the patterns of DNA methylation [55] and mtDNA copy number in naïve, pluripotent, embryonic stem cells [56,57,58] and the changes in DNA methylation that take place as these cells differentiate into mature cell types [59], although it appears that DNA methyation is regulated in a very different manner by the two key processes (the TET and DNMT pathways) [12,58,60]. Importantly, it remains to be determined whether these synchronous changes in mtDNA replication and DNA methylation are interlinked or merely concurrent.

Whilst it has already been argued that there is no active replication taking place which leads to persistent increases in mtDNA copy number prior to gastrulation, it is evident from studies in embryonic stem cells that there are a number of large-scale but short-lived replication events. These events act as checking mechanisms prior to differentiation to ensure that the nuclear and mitochondrial genomes are effectively interacting, and low levels of mtDNA copy number are then restored [61,62]. One key mtDNA replication event takes place on day six of differentiation in mouse embryonic stem cells [61], equivalent to embryonic day six (E7.5). This is consistent with embryonic lethality observed at E7.5 in mouse embryos that are homozygous null for the catalytic subunit of the mtDNA specific polymerase (Polg) [63]. Indeed, failure to replicate mtDNA at this critical time point prevents embryonic stem cells from completing differentiation and is indicative of somatic cells that have been reprogrammed to be embryonic stem-like cells, namely induced pluripotent stem (iPS) cells, that have not undergone complete reprogramming [62]. However, when such cells are treated with DNA demethylation agents, they then have the capability to mediate key stage-specific mtDNA replication events, especially at day 6, and are then able to complete differentiation into mature cell types.

A similar scenario exists in tumor initiating cells in that they do not have the capability to complete differentiation. To this extent, tumor initiating cells initiate the process of differentiation but, due to their extensive hyper-methylation, they are unable to synchronize differentiation with synchronous increases in mtDNA copy number [64]. As a result, they are caught in a ‘mtDNA trap’ where differentiation and mtDNA replication stall [65]. These cells, thus, rely on aerobic glycolysis and proliferate extensively to propagate tumor formation [66]. However, as with incompletely reprogrammed iPS cells, the use of DNA demethylation agents enables tumor initiating cells to complete differentiation and replicate their mtDNA in a manner synchronous with differentiation [67,68].

In relation to mtDNA replication, the mtDNA replication factors are known to be under the control of DNA methylation and in a cell-specific manner that accounts for cell-specific mtDNA copy number [57,68]. Indeed, whilst the majority of factors appear to be DNA methyated to varying degrees, only POLGA and TOP1MT are significantly altered following the use of DNA demethylation agents [57,68]. Furthermore, methylation at exon 2 of POLGA has been shown to be regulated during embryonic stem cell differentiation [57,67] and in the final stages of oocyte maturation [69] and, thus, is also likely during germ cell differentiation. 

## 8. The Importance of mtDNA Replication Efficiency in Differentiating Female Germline Stem Cells

Female germline stem cells can be derived through a number of processes [28]. The population that is present in the ovary, mainly primordial germ cells, are known to mature during oogenesis. However, not all of these will give rise to primordial follicles as many will be eliminated through atresia prior to birth [70] and these outcomes are not related to OXPHOS even though significant increases in mtDNA copy take place [71]. Furthermore, not all follicles are recruited to become the dominant follicle that gives rise to singleton pregnancies. In a number of instances, the oocytes of some women attending assisted reproductive clinics exhibit reduced mtDNA copy number in their metaphase II oocytes [72,73,74]. This most likely relates to the failure of the differentiating oocyte to regulate the replication of mtDNA during oogenesis [69].

Female germline stem cells derived from the ovary of a number of adult species [1,3,75,76], for example egg precursor cells or oogonial stem cells, exhibit many properties similar to adult stem cells in that they express markers associated with early progenitor cells, self-renewal and proliferation and regulators of the cell cycle [1,76,77], although their presence is still deemed to be controversial [78]. Whilst the properties can be species specific, for example the contrast between mouse and pig egg precursor cells [76,77], they exhibit similar differences observed between mouse and human embryonic stem cells where mouse embryonic stem cells demonstrate a more pluripotent, naïver state [79]. Furthermore, egg precursor cells exhibit mitochondrial distribution patterns that are indicative of adult stem cells [80]. They appear to possess oval-like mitochondria [80] that have not formed the complex networks associated with differentiated cells [18]. Indeed, these cell types primarily utilise glycolysis to generate energy with little assistance from OXPHOS. The key factor associated with these mitochondria would be their ability to increase in number as they differentiate towards a mature oocyte that has fertilization capacity. This process would be mediated by nuclear-encoded factors associated with mitochondrial biogenesis that would include the Sirtuin family members SIRT1 and 3; as well as PGC1α, PPARγ, NRF1/2, and the estrogen-related receptor (ERR) α/β [81,82]. Likewise and, in terms of mtDNA, they would need to increase copy number in the final stages of oocyte maturation, mediated through the nuclear-encoded mtDNA replication factors that would include TFAM, mtSSB, Twinkle, POLGA, POLGB, and TOP1MT [49,51]. Failure to do so would likely result in failure to develop to the metaphase II-stage, fertilize, or develop through to the blastocyst stage. This would be a similar outcome for women who exhibited developmental arrest resulting from too few mtDNA copies present at the time fertilization [72,73,74,83], and similar to the mtDNA depletion syndromes associated with mitochondrial disease in somatic tissues [84,85]. These diseases often arise from mutations present in *POLGA* [86] and *TFAM* [87] and, consequently, result in the protein being poorly expressed and a failure to faithfully replicate the mitochondrial genome. 

In human and other mammalian oocytes, decreased expression of *POLGA* has resulted in the failure of oocytes to fertilize [69,88]. This likely arises from levels of DNA methylation regulating the expression of this gene [67] rather than due to mutation as is the case in mitochondrial disease [86]. However, supplementation of poor quality oocytes with extra naïve, oval mitochondria, containing mtDNA, differentially methylated specific CpG sites within the large CpG island in *POLGA* between the metaphase II oocyte and 2-cell embryo stages [69]; and resulted in improved fertilization and blastocyst rates [36]. Consequently, if female germline stem cells are to be used as a source of oocytes in assisted reproduction, it is essential that they adopt the characteristics of the differentiating oocyte and regulate DNA methylation and mtDNA replication events in a synchronous manner to produce viable oocytes.

## 9. The Transmission of mtDNA Mutations and Variants through the Female Germline and mtDNA Disease 

It has been well-established that the female germline harbors variants and mutations that can be transmitted through to the offspring (for an extensive review see [85]). Indeed, it has been argued that the population of mtDNA within the female germline is a distinct, protected population of mitochondrial genomes that do not harbor all of the variants that can be identified in the somatic tissues [89,90,91,92]. This is likely due to the selection, or ‘mitochondrial bottleneck’, events that take place very early during oogenesis to refine or select for specific variants or mutations that are transmitted through the germline [93,94]. Indeed, somatic tissues can harbor spontaneous or de novo variants that more frequently occur in the mitochondrial genome than in the nuclear genome [95] perhaps due to the mode of packaging afforded to the mitochondrial genome [50,96]. Nevertheless, for the pathogenic mtDNA mutations and deletions that give rise to the severe and, sometimes, fatal, multi-systemic mitochondrial diseases, the levels of these rearrangements can be very different in the germline compared to somatic tissues [89,90,91,92]. For example, oocytes can harbor high levels of pathogenic rearrangements that, when prevalent in somatic tissues, can give rise to severe mitochondrial disease. Indeed, 1:200 women are carriers of pathogenic rearrangements [89,97,98], however, the incidence of mitochondrial disease is 1:5000 to 1:10,000 [85]. This clearly suggests that, post-gastrulation, there is selection for and against these rearrangements. However, non-pathogenic rearrangements, which are present in the germline and are found at high levels in mature oocytes, tend to be suppressed in somatic tissues, which suggests a favorable selection of wild type molecules to support fetal development and the well-being of the resultant offspring [99].

In order to maintain these important mitochondrial selection events in female germline stem cells, especially those derived through stem cell technologies, it is essential that these cells harbor rearrangements and variants similar to those present in primordial germ cells and the resultant mature oocyte associated with that particular maternal lineage. Indeed, the use of mtDNA next generation sequencing technology, as with its forerunners, has been extremely useful in identifying maternal ancestral lineages; and can be applied to determine whether putative germline stem cells originate from the pool of progenitor stem cells that give rise to the primordial germ cells. In a study using a mini-pig model derived from a single maternal ancestor that had been characterized for mtDNA rearrangements over several generations [99], egg precursor cells isolated from the ovaries of several females showed a very close alignment to the rearrangements specific to the germline; hence supporting the hypothesis that these cells were of germline origin [100]. The interesting concept to determine in this context is whether the mtDNA profiles of those female germline stem cells derived from embryonic stem cells or through somatic cell reprogramming revert to germline origin not just from a copy number perspective but also through the rearrangements that they harbor. This would answer some key questions: (1) Would the reprogrammed nucleus of the differentiated nucleus, if involved in the selection of rearrangements, select in the same manner as primordial germ cells and potentially egg precursor cells? (2) If not, would this have the propensity to purify or contaminate the female germline with wanted/unwanted variants? (3) Would the rearrangements affect the gene expression profiles of the germline stem cells as is the case for tumor-initiating cells when their mtDNA backgrounds are altered [101]? and (4) the generation of female germline stem cells from human and mouse embryonic stem cells would perhaps help answer the age-old question of whether the later stage characteristics of human embryonic stem cells are really truly pluripotent and have the potential to give rise to germline stem cells. 

## 10. The Use of Female Germline Stem Cells to Overcome Female-Factor Infertility and mtDNA Disease

The ability to isolate egg precursor cells [1,4,102] and to derive female germline stem cells from embryonic [103,104] or induced pluripotent stem cells [105,106] offers considerable hope for infertile couples whose infertility relates to oocyte quality. These cells can be either cultured in vitro to produce mature, metaphase II oocytes and fertilized to generate embryos for transfer to the mother; or transplanted into the recipient’s ovary to promote ovarian function and to increase the likelihood of achieving a pregnancy and a live birth, as so far demonstrated in mouse models [107,108]. They could also be used as a source of mtDNA to promote fertilization outcome for couples who suffer from mtDNA deficiency of the oocyte, namely where there are too few copies of mtDNA to support fertilization outcome [34,36]. This form of autologous mitochondrial supplementation is designed to overcome the ethical and health issues associated with cytoplasmic transfer, where cytoplasm from the oocytes of a young donor has been introduced into the patient’s oocyte and resulted in heteroplasmic offspring and severe associated disorders [109,110,111]. This approach has now been banned by many countries that have embryo regulating authorities. However, in a pig model, the use of autologous mitochondrial supplementation appears to improve embryo development rates and gene expression profiles by the blastocyst stage when mitochondria were isolated from sister oocytes [36]. Nevertheless, a recent study has demonstrated that autologous mitochondrial supplementation using egg precursor cell mitochondria can result in the transgenerational transmission of a heart defect [112]. Whilst this study was conducted in a mouse model, further investigation is required in a large animal model prior to progressing clinical trials. That having been said, a few pregnancies and live births that have been generated using this technology [113,114]. Nevertheless, the use of this technology in a clinical setting should be halted until the procedure has been fully validated in a large animal model with a similar embryo and pathophysiology to that of humans.

**Figure 3:**
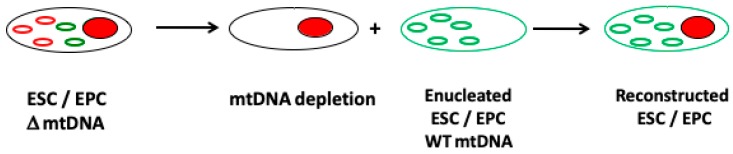
The use of female germline stem cells to overcome mtDNA disease. Female germline stem cells from a carrier of a mtDNA mutation or deletion can be depleted of their mtDNA and fused to an enucleated stem cell harboring unaffected (non-mutated or deleted) mtDNA from a donor source to generate a reconstructed female ‘cybrid’ germline stem cell. The cell can then be proliferated and cultured to the metaphase II stage in readiness for fertilization.

Studies in somatic cell cybrids have shown that a cell’s mtDNA can be replaced with another population of mtDNA [115]. This has also been shown in embryonic stem cell models whereby the chromosomal genome remained unchanged but each cell line possessed a different population of mtDNA [116,117]. These models allow the influence of the mitochondrial genome to be investigated under the same nuclear genome background and demonstrate how mtDNA haploytypes can influence DNA methylation and nuclear gene expression patterns [116,117]. Consequently, an approach to overcome the transmission of mutated or deleted mtDNA from the female carrier could be mediated by replacing her mtDNA with that from a female non-carrier (Figure 3).

**Figure 4:**
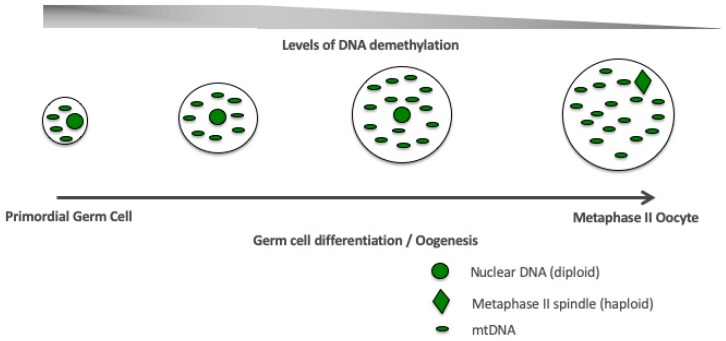
Germ cell differentiation. Stage-specific, synchronized interactions between the nuclear and mitochondrial genomes during germ cell differentiation and oogenesis are required to establish a viable and functional oocyte fit for fertilization (metaphase II oocyte).

If this approach were to be performed in egg precursor or embryonic stem cells derived from the carrier, there would be the potential to generate embryos from mature female germline stem cells. Nevertheless, it is important to recognize that the selection of the most appropriate mtDNA haplotype is essential in order that the resultant cells function effectively and are able to undergo the epigenetic and gene expression changes that take place during germ cell differentiation as well as populating their cytoplasms with sufficient mitochondria and copies of mtDNA (see Figure 4). Indeed, cell function is highly dependent on the compatibility of the nuclear and the mitochondrial genomes with incompatibility, leading to cell dysfunction, poor nuclear gene expression profiles, and perturbed DNA methylation profiles, as demonstrated in embryonic stem cells models of mtDNA haplotypes [101,116,117]; and a model of germ cell differentiation, which resulted in ovarian failure and embryonic lethality [118].

This approach overcomes the necessity for oocyte manipulation and the use of donor oocytes that are currently required for the two technologies associated with mitochondrial donation to produce offspring that would, otherwise, be likely to inherit mitochondrial disease, namely spindle and pronuclear transfer [119,120]. Indeed, the two technologies are also highly dependent on the most appropriate mtDNA haplotype from an unaffected donor oocyte being selected [42]. Furthermore, use of the cybrid approach would allow the interactions of the nuclear and mitochondrial genomes to be established throughout germ cell differentiation, which might not be fully resolved when spindle and pronuclear transfer are performed, given that these technologies are based on the products of end-stage differentiation. However, we are a long way from being able to offer the manipulation of female germline stem cells to treat mitochondrial disease.

## 11. Conclusions

Overall, there is much to discover regarding the role of mitochondria and mtDNA and its replication in female germline stem cells. However, from our knowledge of other closely related cellular systems, for example embryonic and adult stem cells, it is evident that they have significant roles to play. A key facet is the interaction between the nuclear and mitochondrial genomes to regulate mtDNA replication to ensure, during early development, mtDNA copy number is maintained at low levels to promote proliferation but, as differentiation takes place, the copy number needs to increase in an exponential manner (Figure 4). This will ensure that the mature oocyte can provide its mtDNA investment to support subsequent developmental events. Failure would result in failed fertilization or embryo arrest during preimplantation development. These processes are coupled with epigenetic changes and altered gene expression profiles during germ cell differentiation (Figure 4). These events will ultimately determine whether female germline stem cells isolated from the ovary or derived from embryonic stem cells or reprogrammed somatic cells have the potential to generate healthy and metabolically fit offspring and allow their genomes to be modified to prevent the transmission of disease.

## References

[1] Clarkson Y.L., McLaughlin M., Waterfall M., Dunlop C.E., Skehel P.A., Anderson R.A., Telfer E.E. (2018). Initial characterisation of adult human ovarian cell populations isolated by DDX4 expression and aldehyde dehydrogenase activity. Sci. Rep..

[2] Woods D.C., Telfer E.E., Tilly J.L. (2012). Oocyte family trees: Old branches or new stems?. PLoS Genet..

[3] White Y.A., Woods D.C., Takai Y., Ishihara O., Seki H., Tilly J.L. (2012). Oocyte formation by mitotically active germ cells purified from ovaries of reproductive-age women. Nat. Med..

[4] Woods D.C., White Y.A., Tilly J.L. (2013). Purification of oogonial stem cells from adult mouse and human ovaries: An assessment of the literature and a view toward the future. Reprod. Sci..

[5] Duchen M.R. (1999). Contributions of mitochondria to animal physiology: From homeostatic sensor to calcium signalling and cell death. J. Physiol..

[6] Martinez F., Olvera-Sanchez S., Esparza-Perusquia M., Gomez-Chang E., Flores-Herrera O. (2015). Multiple functions of syncytiotrophoblast mitochondria. Steroids.

[7] Sheshadri P., Kumar A. (2016). Managing odds in stem cells: Insights into the role of mitochondrial antioxidant enzyme MnSOD. Free Radic. Res..

[8] Wang Z., Figueiredo-Pereira C., Oudot C., Vieira H.L., Brenner C. (2017). Mitochondrion: A Common Organelle for Distinct Cell Deaths?. Int. Rev. Cell Mol. Biol..

[9] Monlun M., Hyernard C., Blanco P., Lartigue L., Faustin B. (2017). Mitochondria as Molecular Platforms Integrating Multiple Innate Immune Signalings. J. Mol. Biol..

[10] Cloonan S.M., Choi A.M. (2012). Mitochondria: Commanders of innate immunity and disease?. Curr. Opin. Immunol..

[11] West A.P., Shadel G.S., Ghosh S. (2011). Mitochondria in innate immune responses. Nat. Rev. Immunol..

[12] Haseeb A., Makki M.S., Haqqi T.M. (2014). Modulation of ten-eleven translocation 1 (TET1), Isocitrate Dehydrogenase (IDH) expression, alpha-Ketoglutarate (alpha-KG), and DNA hydroxymethylation levels by interleukin-1beta in primary human chondrocytes. J. Biol. Chem..

[13] Martinez-Reyes I., Diebold L.P., Kong H., Schieber M., Huang H., Hensley C.T., Mehta M.M., Wang T., Santos J.H., Woychik R. (2016). TCA Cycle and Mitochondrial Membrane Potential Are Necessary for Diverse Biological Functions. Mol. Cell.

[14] Birky C.W. (1995). Uniparental inheritance of mitochondrial and chloroplast genes: Mechanisms and evolution. Proc. Natl. Acad. Sci. USA.

[15] Van Blerkom J. (2004). Mitochondria in human oogenesis and preimplantation embryogenesis: Engines of metabolism, ionic regulation and developmental competence. Reproduction.

[16] Sathananthan H., Pera M., Trounson A. (2002). The fine structure of human embryonic stem cells. Reprod. Biomed. Online.

[17] Schultz J., Waterstradt R., Kantowski T., Rickmann A., Reinhardt F., Sharoyko V., Mulder H., Tiedge M., Baltrusch S. (2016). Precise expression of Fis1 is important for glucose responsiveness of beta cells. J. Endocrinol..

[18] St John J.C., Ramalho-Santos J., Gray H.L., Petrosko P., Rawe V.Y., Navara C.S., Simerly C.R., Schatten G.P. (2005). The expression of mitochondrial DNA transcription factors during early cardiomyocyte in vitro differentiation from human embryonic stem cells. Cloning Stem Cells.

[19] Chen H., Detmer S.A., Ewald A.J., Griffin E.E., Fraser S.E., Chan D.C. (2003). Mitofusins Mfn1 and Mfn2 coordinately regulate mitochondrial fusion and are essential for embryonic development. J. Cell Biol..

[20] Pfeiffer T., Schuster S., Bonhoeffer S. (2001). Cooperation and competition in the evolution of ATP-producing pathways. Science.

[21] O’Neill L.A., Kishton R.J., Rathmell J. (2016). A guide to immunometabolism for immunologists. Nat. Rev. Immunol..

[22] Moyes C.D., Battersby B.J., Leary S.C. (1998). Regulation of muscle mitochondrial design. J. Exp. Biol..

[23] Trounce I. (2000). Genetic control of oxidative phosphorylation and experimental models of defects. Hum. Reprod..

[24] Quintana-Cabrera R., Mehrotra A., Rigoni G., Soriano M.E. (2017). Who and how in the regulation of mitochondrial cristae shape and function. Biochem. Biophys. Res. Commun..

[25] Bibb M.J., Van Etten R.A., Wright C.T., Walberg M.W., Clayton D.A. (1981). Sequence and gene organization of mouse mitochondrial DNA. Cell.

[26] Ursing B.M., Arnason U. (1998). The complete mitochondrial DNA sequence of the pig (Sus scrofa). J. Mol. Evol..

[27] Nguyen D.H., Jaszczak R.G., Laird D.J. (2019). Heterogeneity of primordial germ cells. Curr. Top. Dev. Biol..

[28] Nikolic A., Volarevic V., Armstrong L., Lako M., Stojkovic M. (2016). Primordial Germ Cells: Current Knowledge and Perspectives. Stem Cells Int..

[29] Cao L., Shitara H., Horii T., Nagao Y., Imai H., Abe K., Hara T., Hayashi J., Yonekawa H. (2007). The mitochondrial bottleneck occurs without reduction of mtDNA content in female mouse germ cells. Nat. Genet..

[30] Cree L.M., Samuels D.C., de Sousa Lopes S.C., Rajasimha H.K., Wonnapinij P., Mann J.R., Dahl H.H., Chinnery P.F. (2008). A reduction of mitochondrial DNA molecules during embryogenesis explains the rapid segregation of genotypes. Nat. Genet..

[31] Wai T., Teoli D., Shoubridge E.A. (2008). The mitochondrial DNA genetic bottleneck results from replication of a subpopulation of genomes. Nat. Genet..

[32] Cotterill M., Harris S.E., Collado Fernandez E., Lu J., Huntriss J.D., Campbell B.K., Picton H.M. (2013). The activity and copy number of mitochondrial DNA in ovine oocytes throughout oogenesis in vivo and during oocyte maturation in vitro. Mol. Hum. Reprod..

[33] St John J.C., Facucho-Oliveira J., Jiang Y., Kelly R., Salah R. (2010). Mitochondrial DNA transmission, replication and inheritance: A journey from the gamete through the embryo and into offspring and embryonic stem cells. Hum. Reprod. Update.

[34] Tilly J.L., Sinclair D.A. (2013). Germline energetics, aging, and female infertility. Cell Metab..

[35] Wu L.L., Russell D.L., Wong S.L., Chen M., Tsai T., St. John J.C., Norman R.J., Febbraio M.A., Carroll J., Robker R.L. (2015). Mitochondrial dysfunction in oocytes of obese mothers; transmission to offspring and reversal by pharmacological ER-stress inhibitors. Development.

[36] Cagnone G.L.M., Tsai T.S., Makanji Y., Matthews P., Gould J., Bonkowski M.S., Elgass K.D., Wong A.S.A., Wu L.E., McKenzie M. (2016). Restoration of normal embryogenesis by mitochondrial supplementation in pig oocytes exhibiting mitochondrial DNA deficiency. Sci. Rep..

[37] Teixeira F.K., Sanchez C.G., Hurd T.R., Seifert J.R., Czech B., Preall J.B., Hannon G.J., Lehmann R. (2015). ATP synthase promotes germ cell differentiation independent of oxidative phosphorylation. Nat. Cell Biol..

[38] Spikings E.C., Alderson J., St John J.C. (2007). Regulated mitochondrial DNA replication during oocyte maturation is essential for successful porcine embryonic development. Biol. Reprod..

[39] Stigliani S., Persico L., Lagazio C., Anserini P., Venturini P.L., Scaruffi P. (2014). Mitochondrial DNA in Day 3 embryo culture medium is a novel, non-invasive biomarker of blastocyst potential and implantation outcome. Mol. Hum. Reprod..

[40] Niwa H., Toyooka Y., Shimosato D., Strumpf D., Takahashi K., Yagi R., Rossant J. (2005). Interaction between Oct3/4 and Cdx2 determines trophectoderm differentiation. Cell.

[41] Houghton F.D. (2006). Energy metabolism of the inner cell mass and trophectoderm of the mouse blastocyst. Differentiation.

[42] St John J. (2014). The control of mtDNA replication during differentiation and development. Biochim. Biophys. Acta.

[43] Sun X., St John J.C. (2016). The role of the mtDNA set point in differentiation, development and tumorigenesis. Biochem. J..

[44] St John J.C., Srirattana K., Tsai T.S., Sun X. (2017). The mitochondrial genome: How it drives fertility. Reprod. Fertil. Dev..

[45] Luo S., Valencia C.A., Zhang J., Lee N.C., Slone J., Gui B., Wang X., Li Z., Dell S., Brown J. (2018). Biparental Inheritance of Mitochondrial DNA in Humans. Proc. Natl. Acad. Sci. USA.

[46] Schwartz M., Vissing J. (2002). Paternal inheritance of mitochondrial DNA. N. Engl. J. Med..

[47] St John J., Sakkas D., Dimitriadi K., Barnes A., Maclin V., Ramey J., Barratt C., De Jonge C. (2000). Failure of elimination of paternal mitochondrial DNA in abnormal embryos. Lancet.

[48] Sutovsky P., Moreno R.D., Ramalho-Santos J., Dominko T., Simerly C., Schatten G. (1999). Ubiquitin tag for sperm mitochondria. Nature.

[49] Kucej M., Butow R.A. (2007). Evolutionary tinkering with mitochondrial nucleoids. Trends Cell Biol..

[50] Farge G., Falkenberg M. (2019). Organization of DNA in Mammalian Mitochondria. Int. J. Mol. Sci..

[51] Falkenberg M., Larsson N.G., Gustafsson C.M. (2007). DNA replication and transcription in mammalian mitochondria. Annu. Rev. Biochem..

[52] Stewart K.R., Veselovska L., Kelsey G. (2016). Establishment and functions of DNA methylation in the germline. Epigenomics.

[53] Smallwood S.A., Kelsey G. (2012). De novo DNA methylation: A germ cell perspective. Trends Genet..

[54] Von Meyenn F., Berrens R.V., Andrews S., Santos F., Collier A.J., Krueger F., Osorno R., Dean W., Rugg-Gunn P.J., Reik W. (2016). Comparative Principles of DNA Methylation Reprogramming during Human and Mouse In Vitro Primordial Germ Cell Specification. Dev. Cell.

[55] Wray J., Kalkan T., Smith A.G. (2010). The ground state of pluripotency. Biochem. Soc. Trans..

[56] Facucho-Oliveira J.M., St John J.C. (2009). The relationship between pluripotency and mitochondrial DNA proliferation during early embryo development and embryonic stem cell differentiation. Stem Cell Rev..

[57] Kelly R.D., Mahmud A., McKenzie M., Trounce I.A., St John J.C. (2012). Mitochondrial DNA copy number is regulated in a tissue specific manner by DNA methylation of the nuclear-encoded DNA polymerase gamma A. Nucleic Acids Res..

[58] Ross S.E., Bogdanovic O. (2019). TET enzymes, DNA demethylation and pluripotency. Biochem. Soc. Trans..

[59] Wiehle L., Thorn G.J., Raddatz G., Clarkson C.T., Rippe K., Lyko F., Breiling A., Teif V.B. (2019). DNA (de)methylation in embryonic stem cells controls CTCF-dependent chromatin boundaries. Genome Res..

[60] King A.D., Huang K., Rubbi L., Liu S., Wang C.Y., Wang Y., Pellegrini M., Fan G. (2016). Reversible Regulation of Promoter and Enhancer Histone Landscape by DNA Methylation in Mouse Embryonic Stem Cells. Cell Rep..

[61] Facucho-Oliveira J.M., Alderson J., Spikings E.C., Egginton S., St John J.C. (2007). Mitochondrial DNA replication during differentiation of murine embryonic stem cells. J. Cell Sci..

[62] Kelly R.D., Sumer H., McKenzie M., Facucho-Oliveira J., Trounce I.A., Verma P.J., St John J.C. (2013). The effects of nuclear reprogramming on mitochondrial DNA replication. Stem Cell Rev..

[63] Hance N., Ekstrand M.I., Trifunovic A. (2005). Mitochondrial DNA polymerase gamma is essential for mammalian embryogenesis. Hum. Mol. Genet..

[64] Dickinson A., Yeung K.Y., Donoghue J., Baker M.J., Kelly R.D., McKenzie M., Johns T.G., St John J.C. (2013). The regulation of mitochondrial DNA copy number in glioblastoma cells. Cell Death Differ..

[65] Lee W.T., John J.S. (2015). The control of mitochondrial DNA replication during development and tumorigenesis. Ann. N. Y. Acad. Sci..

[66] Vander Heiden M.G., Cantley L.C., Thompson C.B. (2009). Understanding the Warburg effect: The metabolic requirements of cell proliferation. Science.

[67] Lee W., Johnson J., Gough D.J., Donoghue J., Cagnone G.L.M., Vaghjiani V., Brown K.A., Johns T.G., St. John J.C. (2015). Mitochondrial DNA copy number is regulated by DNA Methylation and demethylation of POLGA in stem and cancer cells and their differentiated progeny. Cell Death Dis..

[68] Sun X., Johnson J., St John J.C. (2018). Global DNA methylation synergistically regulates the nuclear and mitochondrial genomes in glioblastoma cells. Nucleic Acids Res..

[69] Tsai T.S., Tyagi S., St John J.C. (2018). The molecular characterisation of mitochondrial DNA deficient oocytes using a pig model. Hum. Reprod..

[70] Faddy M.J., Gosden R.G., Gougeon A., Richardson S.J., Nelson J.F. (1992). Accelerated disappearance of ovarian follicles in mid-life: Implications for forecasting menopause. Hum. Reprod..

[71] Shoubridge E.A., Wai T. (2007). Mitochondrial DNA and the mammalian oocyte. Curr. Top. Dev. Biol..

[72] Santos T.A., El Shourbagy S., St John J.C. (2006). Mitochondrial content reflects oocyte variability and fertilization outcome. Fertil. Steril..

[73] May-Panloup P., Chretien M.F., Jacques C., Vasseur C., Malthiery Y., Reynier P. (2005). Low oocyte mitochondrial DNA content in ovarian insufficiency. Hum. Reprod..

[74] Reynier P., May-Panloup P., Chretien M.F., Morgan C.J., Jean M., Savagner F., Barriere P., Malthiery Y. (2001). Mitochondrial DNA content affects the fertilizability of human oocytes. Mol. Hum. Reprod..

[75] Johnson J., Canning J., Kaneko T., Pru J.K., Tilly J.L. (2004). Germline stem cells and follicular renewal in the postnatal mammalian ovary. Nature.

[76] Bui H.T., Van Thuan N., Kwon D.N., Choi Y.J., Kang M.H., Han J.W., Kim T., Kim J.H. (2014). Identification and characterization of putative stem cells in the adult pig ovary. Development.

[77] Xie W., Wang H., Wu J. (2014). Similar morphological and molecular signatures shared by female and male germline stem cells. Sci. Rep..

[78] Zarate-Garcia L., Lane S.I., Merriman J.A., Jones K.T. (2016). FACS-sorted putative oogonial stem cells from the ovary are neither DDX4-positive nor germ cells. Sci. Rep..

[79] Ginis I., Luo Y., Miura T., Thies S., Brandenberger R., Gerecht-Nir S., Amit M., Hoke A., Carpenter M.K., Itskovitz-Eldor J. (2004). Differences between human and mouse embryonic stem cells. Dev. Biol..

[80] Parte S., Bhartiya D., Patel H., Daithankar V., Chauhan A., Zaveri K., Hinduja I. (2014). Dynamics associated with spontaneous differentiation of ovarian stem cells in vitro. J. Ovarian Res..

[81] Aquilano K., Vigilanza P., Baldelli S., Pagliei B., Rotilio G., Ciriolo M.R. (2010). Peroxisome proliferator-activated receptor gamma co-activator 1alpha (PGC-1alpha) and sirtuin 1 (SIRT1) reside in mitochondria: Possible direct function in mitochondrial biogenesis. J. Biol. Chem..

[82] Scarpulla R.C., Vega R.B., Kelly D.P. (2012). Transcriptional integration of mitochondrial biogenesis. Trends Endocrinol. Metab..

[83] Cohen J., Scott R., Schimmel T., Levron J., Willadsen S. (1997). Birth of infant after transfer of anucleate donor oocyte cytoplasm into recipient eggs. Lancet.

[84] Rahman S., Poulton J. (2009). Diagnosis of mitochondrial DNA depletion syndromes. Arch. Dis. Child.

[85] McFarland R., Taylor R.W., Turnbull D.M. (2007). Mitochondrial disease--its impact, etiology, and pathology. Curr. Top. Dev. Biol..

[86] Copeland W.C. (2008). Inherited mitochondrial diseases of DNA replication. Annu. Rev. Med..

[87] Guo J., Zheng L., Liu W., Wang X., Wang Z., French A.J., Kang D., Chen L., Thibodeau S.N. (2011). Frequent truncating mutation of TFAM induces mitochondrial DNA depletion and apoptotic resistance in microsatellite-unstable colorectal cancer. Cancer Res..

[88] Boucret L., Chao de la Barca J.M., Moriniere C., Desquiret V., Ferre-L’Hotellier V., Descamps P., Marcaillou C., Reynier P., Procaccio V., May-Panloup P. (2015). Relationship between diminished ovarian reserve and mitochondrial biogenesis in cumulus cells. Hum. Reprod..

[89] Elliott H.R., Samuels D.C., Eden J.A., Relton C.L., Chinnery P.F. (2008). Pathogenic mitochondrial DNA mutations are common in the general population. Am. J. Hum. Genet..

[90] Stewart J.B., Freyer C., Elson J.L., Wredenberg A., Cansu Z., Trifunovic A., Larsson N.G. (2008). Strong purifying selection in transmission of mammalian mitochondrial DNA. PLoS Biol..

[91] Fan W., Waymire K.G., Narula N., Li P., Rocher C., Coskun P.E., Vannan M.A., Narula J., Macgregor G.R., Wallace D.C. (2008). A mouse model of mitochondrial disease reveals germline selection against severe mtDNA mutations. Science.

[92] Samuels D.C., Li C., Li B., Song Z., Torstenson E., Boyd Clay H., Rokas A., Thornton-Wells T.A., Moore J.H., Hughes T.M. (2013). Recurrent tissue-specific mtDNA mutations are common in humans. PLoS Genet..

[93] Olivo P.D., Van de Walle M.J., Laipis P.J., Hauswirth W.W. (1983). Nucleotide sequence evidence for rapid genotypic shifts in the bovine mitochondrial DNA D-loop. Nature.

[94] Johnston I.G., Burgstaller J.P., Havlicek V., Kolbe T., Rulicke T., Brem G., Poulton J., Jones N.S. (2015). Stochastic modelling, Bayesian inference, and new in vivo measurements elucidate the debated mtDNA bottleneck mechanism. Elife.

[95] Li H., Slone J., Fei L., Huang T. (2019). Mitochondrial DNA Variants and Common Diseases: A Mathematical Model for the Diversity of Age-Related mtDNA Mutations. Cells.

[96] Kaufman B.A., Durisic N., Mativetsky J.M., Costantino S., Hancock M.A., Grutter P., Shoubridge E.A. (2007). The mitochondrial transcription factor TFAM coordinates the assembly of multiple DNA molecules into nucleoid-like structures. Mol. Biol. Cell.

[97] Manwaring N., Jones M.M., Wang J.J., Rochtchina E., Howard C., Mitchell P., Sue C.M. (2007). Population prevalence of the MELAS A3243G mutation. Mitochondrion.

[98] Vandebona H., Mitchell P., Manwaring N., Griffiths K., Gopinath B., Wang J.J., Sue C.M. (2009). Prevalence of mitochondrial 1555A-->G mutation in adults of European descent. N. Engl. J. Med..

[99] Cagnone G., Tsai T.S., Srirattana K., Rossello F., Powell D.R., Rohrer G., Cree L., Trounce I.A., St John J. (2016). Segregation of Naturally Occurring Mitochondrial DNA Variants in a Mini-pig Model. Genetics.

[100] Tsai T.S., Johnson J., White Y., John J.C. (2017). The molecular characterization of porcine egg precursor cells. Oncotarget.

[101] Lee W.T.Y., Cain J.E., Cuddihy A., Johnson J., Dickinson A., Yeung K.Y., Kumar B., Johns T.G., Watkins D.N., Spencer A. (2016). Mitochondrial DNA plasticity is an essential inducer of tumorigenesis. Cell Death Discov..

[102] Clarkson Y.L., Weatherall E., Waterfall M., McLaughlin M., Lu H., Skehel P.A., Anderson R.A., Telfer E.E. (2019). Extracellular Localisation of the C-Terminus of DDX4 Confirmed by Immunocytochemistry and Fluorescence-Activated Cell Sorting. Cells.

[103] Zhou Q., Wang M., Yuan Y., Wang X., Fu R., Wan H., Xie M., Liu M., Guo X., Zheng Y. (2016). Complete Meiosis from Embryonic Stem Cell-Derived Germ Cells In Vitro. Cell Stem Cell.

[104] Hikabe O., Hamazaki N., Nagamatsu G., Obata Y., Hirao Y., Hamada N., Shimamoto S., Imamura T., Nakashima K., Saitou M. (2016). Reconstitution in vitro of the entire cycle of the mouse female germ line. Nature.

[105] Hayashi K., Ohta H., Kurimoto K., Aramaki S., Saitou M. (2011). Reconstitution of the mouse germ cell specification pathway in culture by pluripotent stem cells. Cell.

[106] Hayashi K., Ogushi S., Kurimoto K., Shimamoto S., Ohta H., Saitou M. (2012). Offspring from oocytes derived from in vitro primordial germ cell-like cells in mice. Science.

[107] Zou K., Yuan Z., Yang Z., Luo H., Sun K., Zhou L., Xiang J., Shi L., Yu Q., Zhang Y. (2009). Production of offspring from a germline stem cell line derived from neonatal ovaries. Nat. Cell Biol..

[108] Wu C., Xu B., Li X., Ma W., Zhang P., Chen X., Wu J. (2017). Tracing and Characterizing the Development of Transplanted Female Germline Stem Cells In Vivo. Mol. Ther..

[109] Barritt J.A., Brenner C.A., Malter H.E., Cohen J. (2001). Rebuttal: Interooplasmic transfers in humans. Reprod. Biomed. Online.

[110] Brenner C.A., Barritt J.A., Willadsen S., Cohen J. (2000). Mitochondrial DNA heteroplasmy after human ooplasmic transplantation. Fertil. Steril..

[111] Acton B.M., Lai I., Shang X., Jurisicova A., Casper R.F. (2007). Neutral mitochondrial heteroplasmy alters physiological function in mice. Biol. Reprod..

[112] St John J.C., Makanji Y., Johnson J.L., Tsai T.S., Lagondar S., Rodda F., Sun X., Pangestu M., Chen P., Temple-Smith P. (2019). The transgenerational effects of oocyte mitochondrial supplementation. Sci. Rep..

[113] Fakih M., El Shmoury M., Szeptycki J., dela Cruz D.B., Lux C., Verjee S., Burgess C.M., Cohn G.M., Casper R.F. (2015). The AUGMENTSM Treatment: Physician Reported Outcomes of the Initial Global Patient Experience. JFIV Reprod. Med. Genet..

[114] Oktay K., Baltaci V., Sonmezer M., Turan V., Unsal E., Baltaci A., Aktuna S., Moy F. (2015). Oogonial Precursor Cell-Derived Autologous Mitochondria Injection to Improve Outcomes in Women with Multiple IVF Failures Due to Low Oocyte Quality: A Clinical Translation. Reprod. Sci..

[115] Trounce I., Wallace D.C. (1996). Production of transmitochondrial mouse cell lines by cybrid rescue of rhodamine-6G pre-treated L-cells. Somat. Cell Mol. Genet..

[116] Kelly R.D., Rodda A.E., Dickinson A., Mahmud A., Nefzger C.M., Lee W., Forsythe J.S., Polo J.M., Trounce I.A., McKenzie M. (2013). Mitochondrial DNA haplotypes define gene expression patterns in pluripotent and differentiating embryonic stem cells. Stem Cells.

[117] Lee W.T., Sun X., Tsai T.S., Johnson J.L., Gould J.A., Garama D.J., Gough D.J., McKenzie M., Trounce I.A., St John J.C. (2017). Mitochondrial DNA haplotypes induce differential patterns of DNA methylation that result in differential chromosomal gene expression patterns. Cell Death Discov..

[118] Zhang C., Montooth K.L., Calvi B.R. (2017). Incompatibility between mitochondrial and nuclear genomes during oogenesis results in ovarian failure and embryonic lethality. Development.

[119] Tachibana M., Sparman M., Sritanaudomchai H., Ma H., Clepper L., Woodward J., Li Y., Ramsey C., Kolotushkina O., Mitalipov S. (2009). Mitochondrial gene replacement in primate offspring and embryonic stem cells. Nature.

[120] Craven L., Tuppen H.A., Greggains G.D., Harbottle S.J., Murphy J.L., Cree L.M., Murdoch A.P., Chinnery P.F., Taylor R.W., Lightowlers R.N. (2010). Pronuclear transfer in human embryos to prevent transmission of mitochondrial DNA disease. Nature.

